# Comparison of screening strategies for Lynch syndrome in patients with newly diagnosed endometrial cancer: a prospective cohort study in China

**DOI:** 10.1186/s40880-019-0388-2

**Published:** 2019-07-15

**Authors:** Xiaopei Chao, Lei Li, Ming Wu, Shuiqing Ma, Xianjie Tan, Sen Zhong, Yalan Bi, Jinghe Lang

**Affiliations:** 1 Department of Obstetrics and Gynecology, Peking Union Medical College Hospital, Peking Union Medical College & Chinese Academy of Medical Science, Shuaifuyuan No. 1, Dongcheng District, Beijing, 100730 P.R. China; 2 Department of Pathology, Peking Union Medical College Hospital, Peking Union Medical College & Chinese Academy of Medical Science, Beijing, 100730 P.R. China

**Keywords:** Endometrial carcinoma, Lynch syndrome, Immunohistochemistry, Microsatellite instability, Germline mutations, DNA mismatch repair genes

## Abstract

**Background:**

The prevalence of Lynch syndrome and screening strategies for this disorder in Chinese patients with endometrial cancer have seldom been investigated. Such data would be essential for the screening, prevention, genetic counseling, and treatment of Lynch syndrome. The purpose of this prospective study was to determine the accuracy of the mismatch repair (MMR) protein immunohistochemistry (IHC), microsatellite instability (MSI) test, and clinical diagnostic criteria in screening for Lynch syndrome-associated endometrial cancer (LS-EC) in a prospective Chinese cohort.

**Methods:**

All patients with newly diagnosed endometrial cancer (EC) were evaluated using clinical diagnostic criteria (Amsterdam II criteria and the revised Bethesda guidelines), MSI test, and IHC of MMR proteins in tumor tissues. For all patients, the screening results were compared with results of germline sequencing for pathogenic variants of MMR genes.

**Results:**

Between December 2017 and August 2018, a total of 111 unselected patients with newly diagnosed EC were enrolled. Six patients (5.4%) harbored a pathogenic germline mutation of MMR genes: 1 had a mutation in MutL homolog 1 (*MLH1*), 2 in MutS homolog 2 (*MSH2*), and 3 in MutS homolog 6 (*MSH6*). The sensitivity, specificity, positive predictive value (PPV), and negative predictive value (NPV) for identifying LS-EC were 33.3%, 88.6%, 14.3%, and 95.9%, for the clinical criteria, 66.7%, 75.0%, 14.3%, and 97.3% for IHC of MMR proteins, 100%, 89.9%, 33.3%, and 100% for MSI test, and 100%, 72.4%, 20.0% and 100% for combined IHC and MSI test, respectively. The combination of IHC and MSI test had higher sensitivity and PPV than the clinical criteria (*p* = 0.030). MSI test and IHC were highly concordant for LS-EC screening (73/77, 94.8%).

**Conclusion:**

The accuracy of the combination of IHC of MMR proteins and MSI test for screening LS among Chinese patients with EC was superior to that of the clinical criteria.

*Trial registration* NCT03291106. Registered on September 25, 2017

## Background

Lynch syndrome (LS) is an autosome-dominant, inherited, cancer susceptibility syndrome characterized by a high risk of malignancies, including colorectal (lifetime risk of 52–82%) [1], endometrial (lifetime risk of 25–60%) [2], and ovarian (lifetime risk of 4–12%) malignancies [3, 4]. The diagnosis of LS is based on the identification of germline mutations in the DNA mismatch repair (MMR) genes MutL homolog 1 (*MLH1*), MutS homolog 2 (*MSH2*), MutS homolog 6 (*MSH6*), PMS1 homolog 2 (*PMS2*), and/or epithelial cell adhesion molecule (*EPCAM*). The loss of DNA MMR leads to genomic instability by facilitating the accumulation of somatic mutations in various sequences. Endometrial cancer (EC) is the most common sentinel cancer in LS patients [5, 6]. Screening and diagnosing LS in EC patients is of great importance for affected individuals and their relatives, who would benefit from genetic counseling and enhanced surveillance [7, 8].

Screening strategies for LS-associated colorectal carcinomas have been studied thoroughly [9, 10]. However, screening for LS-associated EC (LS-EC) is controversial, as no consensus has been reached on the strategies, upper age threshold, or cost-effectiveness of screening [11, 12]. Historically, before the era of next-generation sequencing (NGS), LS screening methods have consisted of clinical diagnostic criteria based on individual and family cancer histories; these methods are relatively inexpensive. The most commonly used screening tools are the Amsterdam II clinical criteria [13] and the revised Bethesda guidelines [14]. However, the disadvantages of clinical criteria are the requirement for high accuracy in collecting individual and family histories and the actual low accuracy [15]. Furthermore, the traditional clinical schemas for LS screening are centered on the colon and have been shown to perform poorly at identifying LS in patients with gynecological malignancies [16]. In addition, these criteria are highly complex and poorly implemented in clinical practice. Therefore, the exploration of more efficient screening strategies for LS among EC patients is needed [17].

Universal molecular screening for LS in all newly diagnosed EC patients has been carried out in numerous centers in Europe [11] and the United States [12]. The National Comprehensive Cancer Network (NCCN) guidelines recommend that all patients with newly diagnosed EC be tested for loss of MMR function via immunohistochemical (IHC) and/or microsatellite instability (MSI) analysis independent of the clinical criteria [18]. This screening method is cost-effective and, at the same time, can ensure sensitivity and specificity [19, 20]. The Austrian Arbeitsgemeinschaft für Gynäkologische Onkologie (AGO) recommends IHC tissue screening for LS in type I and type II ECs among all patients below the age of 70 years and among all patients with endometrioid or clear cell ovarian cancers regardless of age [21]. However, there are few reports on the accuracy, strength, and limitations of these strategies when they are applied in Chinese EC patients. Identifying EC-related genetic factors would promote the development of genetic detection methods for carriers of potential EC-related mutations, potentially improving the health of women by establishing an innovative strategy for screening patients with hereditary carcinoma [22].

The objective of the present study was to determine the accuracy of IHC of MMR proteins, MSI test, and clinical criteria in screening for LS in a prospective cohort of Chinese EC patients. The results of screening were compared with those of universal germline sequencing for pathogenic variants of MMR genes.

## Materials and methods

### Ethics and registration

This study was an interim analysis for the project “Cohort Study of Universal Screening for Lynch Syndrome in Chinese Patients with Endometrial Cancer”, which was approved by the Institutional Review Board of Peking Union Medical College Hospital (Registration no. JS-1370). The registration number in *clinicaltrials.gov* is NCT03291106. The Chinese Human Genetic Resources Management Office of the National Ministry of Science and Technology has approved this study (Registration no. [2018] 522, http://www.most.gov.cn/bszn/new/rlyc/jgcx/index.htm). The enrollment will end by the year 2020. All patients provided consent before enrollment.

### Patient population

All patients newly diagnosed with EC since December 2017 in the Department of Obstetrics & Gynecology of the Peking Union Medical College Hospital were enrolled. Detailed epidemiological, clinical, and pathological data were prospectively collected. Patients with recurrent carcinoma and synchronous carcinomas were excluded.

### Screening protocols

**Fig. 1:**
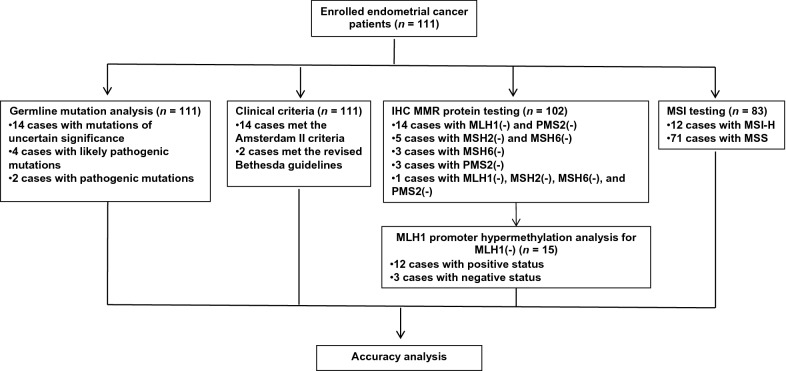
A flow diagram of the study. IHC: immunohistochemistry; MMR: mismatch repair; MLH1: MutL homolog 1; PMS2: PMS1 homolog 2; MSH2: MutS homolog 2; MSH6: MutS homolog 6; MSI: microsatellite instability; MSI-H: high-frequency MSI; MSS: microsatellite stable

The screening protocols consisted of analysis of clinical criteria, IHC of MMR proteins, and MSI test, which were conducted for all patients if possible (Fig. 1). The costs of IHC of MMR proteins, MSI test, and germline sequencing have been reported by Peking Union Medical College Hospital to be 1000 RMB yuan (approximately 143 US dollars), 1500 RMB yuan (214 US dollars), and 3500 RMB yuan (500 US dollars) per patient, respectively. These costs will be covered by the Chinese Academy of Medical Sciences Initiative for Innovative Medicine (CAMS-2017-I2M-1-002) and the National Science-Technology Support Plan Projects (2015BAI13B04). The funders had no role in study design, data collection and analysis, decision to publish, or preparation of the manuscript. Peripheral blood samples were collected right before surgery, whereas bulky uterine tissues resected during hysterectomy were saved for IHC and MSI. No neoadjuvant therapy was administered in this study.

#### Clinical criteria

The patients’ family information was collected by two medical staff members, and their diseases were staged according to the International Federation of Gynecology and Obstetrics (FIGO) staging system. All patients’ records were evaluated according to the Amsterdam II clinical criteria [13] and the revised Bethesda guidelines [14]. Patients meeting either set of criteria were considered to have suspected LS-EC.

#### IHC of MMR proteins

IHC staining of proteins (MLH1, MSH2, MSH6, and PMS2) was performed on 4-µm-thick, formalin-fixed, paraffin-embedded sections of newly diagnosed EC tissues from these patients using a BenchMark ULTRA autostainer, version 12.3 (Ventana Medical Systems, New York, NY, USA) and the ready-to-use Ventana MMR IHC Panel with anti-MLH1 mouse monoclonal antibody (M1, 1:20 dilution), anti-MSH2 mouse monoclonal antibody (G219-1129, 1:100 dilution), anti-MSH6 rabbit monoclonal antibody (SP93, 1:1000 dilution), and anti-PMS2 mouse monoclonal antibody (A16-4, 1:100 dilution) from Roche Applied Science (New York, NY, USA) according to the manufacturer’s recommendations. Normal endometrial and/or myometrial tissues collected at least 3 cm away from the tumor margin were used as a normal control, and the results of IHC staining were examined by two independent pathologists. Intact IHC staining of MMR proteins was defined as positive staining of all MLH1, MSH2, MSH6, and PMS2 proteins, while deficient IHC staining was defined as negative staining of any of these four proteins. For deficient MLH1 expression, the methylation status of the MLH1 promoter was determined with methylation-specific multiplex ligation-dependent probe amplification (MS-MLPA) using the SALSA MS-MLPA Kit ME011-A1 (MRC-Holland, Amsterdam, the Netherlands) as reported by van Lier et al. [23].

#### MSI test

DNA was extracted from tumor tissues using a TIANamp FFPE DNA Kit (TIANGEN Co. Ltd., Beijing, China) according to the manufacturer’s directions. A multiplex polymerase chain reaction (PCR)-capillary electrophoresis MSI detection kit (Sinomdgene Co. Ltd., Beijing, China) was used to perform microsatellite analysis of six mononucleotide repeat markers (BAT-25, BAT-26, NR-21, NR-24, NR-27, and MONO-27) for MSI determination, one sex-determination marker (Amel site) and two pentanucleotide repeat markers (Penta C and Penta D) for identification of the tumor tissues. An internal lane size standard (ROX-500) was added to the amplified samples to ensure accurate allele sizing. All other markers were amplified in 20 µL PCR resolution consisting of 10 µL Master Mix (Sinomdgene), 8 µL primer mixture (Sinomdgene), and 2 µL DNA. The PCR products were separated by capillary electrophoresis using an Applied Biosystems^®^ 3130 or 3500 Dx genetic analyzer (Los Angeles, CA, USA), and the output data were analyzed with the GeneMapper^®^ software (Applied Biosystems) to determine the MSI status of the test samples. Panels and matched files were created to facilitate analysis using the GeneMapper^®^ software. According to the recommendations of the National Cancer Institute [24], tumors with 2 or more of the tested markers to be instable were classified as high-frequency MSI (MSI-H); tumors with less than 2 of these markers to be instable, low-frequency MSI (MSI-L); and tumors without instability markers, microsatellite stable (MSS). MSI-H were designated MSI-positive, whereas MSI-L and MSS were designated MSI-negative.

### Next-generation sequencing for germline mutation analysis

Germline mutation sequencing was performed for all patients. Genomic DNA was extracted from peripheral blood and quantified using a Qubit Instruments device (Life Technologies, Eugene, OR, USA). The extraction was assessed as unsuccessful when the total concentration of the extracted genomic DNA was lower than 25 ng. DNA fragmentation was evaluated by agarose gel electrophoresis. A TILLING PCR amplifier sequence (tPAS) library was constructed and quantified by qPCR. Then, the exons of the targeted genes—*MLH1*, *MSH2*, *MSH6*, *PMS2*, and *EPCAM*—were analyzed on the NextSeq CN500 platform (Hangzhou BerryGenomics Diagnostics Technology Co., Ltd, Hangzhou, Zhejiang, China), and the mutation types included single-nucleotide polymorphisms (SNPs) and insertion-deletions (indels). The data were analyzed using the Verita Trekker^®^ Enliven^®^ Genotypic Interpretation system (Berry Genomics Corporation, Beijing, China). The results for the sequence variants were classified according to the American College of Medical Genetics and Genomics (ACMG) guidelines [25]. If the results were interpreted to indicate a pathogenic/likely pathogenic mutation, the NGS data were confirmed by detecting the mutated gene via Sanger sequencing.

### Statistical analyses

Statistical analyses were performed using the software SPSS, version 22.0 (SPSS Inc., Chicago, IL, USA). Normally distributed continuous variables were summarized as means and standard deviations (SDs), and non-normally distributed continuous variables were summarized as medians, ranges, and interquartile ranges. The *t*-test was used for continuous variables, and the Chi-squared test or Fisher’s exact test was used for categorical variables. Exact binomial 95% confidence intervals (CIs) were calculated to estimate sensitivity, specificity, positive predictive value (PPV), and negative predictive value (NPV) of all screening methods. The McNemar test was used to compare sensitivity values. All analyses were two-tailed, and a *p* value of < 0.05 was considered significant. MSI-H in combination with MMR protein-deficient and MSS in combination with MMR protein-intact were defined as concordance. Any other conditions were defined as disconcordance.

## Results

### Demographic data of the study population

Our study comprised of 112 patients with EC; 1 with pathologically identified primary cervical tumor was subsequently excluded. The demographic and clinicopathological characteristics of the remaining 111 patients are shown in Table 1. The mean and median ages of the study cohort were 55.7 (SD, 9.7) years and 55 (range 31–82) years. Eighty-seven (78.4%) and 24 (21.6%) cases were type I and type II EC, respectively. According to the FIGO staging system, 87 (78.4%), 19 (17.1%), and 4 (3.6%) patients were in stage I, stage III, and stage IV, respectively, none had stage II disease. One patient refused retroperitoneal lymphadenectomy; therefore, the disease stage of that patient was unknown. Twenty-seven (24.3%) patients had involvement of the lower uterine segment.

**Table 1:** Demographic and clinicopathologic characteristics of the 111 unselected patients with endometrial cancer

Characteristic	Total	Germline MMR gene mutation			IHC MMR protein			MSI		
		Absence	Presence	*p* value	Intact	Deficient	*p* value	Negative	Positive	*p* value
Total (cases)	111	105	6		74	28		71	12	
Age (years; median [range])		56.00 (31–82)	54.00 (48–57)	0.440	56.50 (32–82)	53.50 (31–73)	0.102	57.00 (32–82)	55.00 (40–73)	0.658
Gravidity [median (range)]		2 (0–7)	2 (0–5)	0.374	2 (0–5)	2 (0–7)	0.437	2 (0–7)	2 (0–5)	*0.020*
Parity (median, range)		1 (0–4)	1 (0–2)	0.779	1 (0–4)	1 (0–2)	0.498	1 (0–4)	1 (0–3)	0.189
BMI (kg/m^2^; median [range])		24.98 (19–32)	21.60 (19–23)	*0.010*	25.66 (19–32)	24.27 (20–32)	0.622	24.98 (19–32)	23.87 (19–31)	0.343
Involvement of the lower uterine segment [cases (%)]	27	24 (22.9)	3 (50.0)	0.132	17 (23.0)	9 (32.1)	0.343	14 (19.7)	4 (33.3)	0.290
Type of EC [cases (%)]				0.189			0.983			0.937
Type I	87	81 (77.1)	6 (100.0)		58 (78.4)	22 (78.6)		54 (76.1)	9 (75.0)	
Type II^a^	24	24 (22.9)	0 (0)		16 (21.6)	6 (21.4)		17 (23.9)	3 (25.0)	
FIGO stage [cases (%)]^b^				*0.012*			0.266			0.354
FIGO I	87	85 (81.0)	2 (33.3)		61 (82.4)	19 (67.9)		60 (84.5)	9 (75.0)	
FIGO III	19	15 (14.3)	4 (66.7)		11 (14.9)	8 (28.6)		8 (11.3)	3 (25.0)	
FIGO IV	4	4 (3.8)	0 (0)		2 (2.7)	1 (3.6)		3 (4.2)	0 (0)	
Unknown	1	1 (1.0)	0 (0)		0 (0)	0 (0)		0 (0)	0 (0)	

Italic values indicate significance of *p* value (*p* < 0.05)

MMR: mismatch repair; IHC: immunohistochemistry; MSI: microsatellite instability; SD: standard deviation; BMI: body mass index; EC: endometrial carcinoma; FIGO: International Federation of Gynecology and Obstetrics

^a^Among 24 cases of type II endometrial cancer, there were nine cases of the serous subtype, three of clear cell carcinoma, four of carcinosarcoma, one of undifferentiated carcinoma, and seven cases of mixed carcinoma (four cases of endometrioid and clear cell carcinomas, two cases of the endometrioid and serous subtypes, and one case of endometrioid subtype and carcinosarcoma)

^b^No patients had FIGO II disease

### Germline MMR mutation

All 111 patients had definitive germline mutation sequencing data. Among them, 14 (12.6%) had mutations of uncertain significance, 4 (3.6%) had likely pathogenic mutations, and 2 (1.8%) had pathogenic mutations (Table 2). Six (5.4%) patients were ultimately diagnosed with LS-EC with a mutation in one of the MMR genes: 1 in *MLH1*, 2 in *MSH2*, and 3 in *MSH6*. The cumulative cases of LS among the EC patients were 2 (6.3%), 6 (8.6%), and 6 (5.9%) before the ages of 50, 60, and 70, respectively. No patients > 70 years of age had LS. Compared with patients harboring no MMR gene mutations, those with MMR gene mutations (LS) had lower BMI (*p *= 0.013), and the presence of MMR gene mutations was strongly associated with advanced lesions (*p *= 0.012) (Table 1).

**Table 2:** The results of germline mutation sequencing, IHC of MMR proteins, and MSI test for the 20 patients with MMR gene mutations

Case no.	Germline MMR gene mutation^a^							IHC of MMR proteins				MSI	Amsterdam II criteria	Revised Bethesda guidelines
	Gene	Transcript	Gene region	Nucleotide	Amino acid	Function change	Mutation type	MLH1	MSH2	MSH6	PMS2			
1	*MSH6*	NM_000179.2	Exon 4	c.C742T	p.Arg248Ter	Nonsense	Pathogenic	(+)	(+)	(−)	(+)	NA	(−)	(−)
2	*MSH6*	NM_000179.2	Exon 4	c.C3103T	p.Arg1035Ter	Nonsense	Pathogenic	(+)	(+)	(+)	(+)	MSI-H	(−)	(−)
3	*MSH2*	NM_000251.2	Exon 11	c.1677_1680delAAAT	p.Asn560Lysfs	Deletion	Likely pathogenic	(+)	(−)	(−)	(+)	NA	(+)	(+)
4	*MLH1*	NM_000249.3	Exon 12	c.1393dupA	p.Thr465Asnfs	Insertion	Likely pathogenic	(−)	(+)	(+)	(−)	MSI-H	(+)	(+)
5	*MSH2*	NM_000251.2	Exon 12	c.1813delG	p.Val605Leufs	Deletion	Likely pathogenic	(+)	(+)	(+)	(+)	MSI-H	(−)	(−)
6	*MSH6*	NM_000179.2	Exon 4	c.2598_2602delAGTAA	p.Lys866Asnfs	Deletion	Likely pathogenic	(+)	(+)	(−)	(+)	MSI-H	(−)	(−)
7	*MSH6*	NM_000179.2	Exon 4	c.A1828G	p.Lys610Glu	Missense	Uncertain significance	(+)	(+)	(+)	(+)	MSS	(−)	(−)
8	*PMS2*	NM_000535.6	Exon 14	c.G2438A	p.Arg813Gln	Missense	Uncertain significance	(+)	(+)	(+)	(+)	MSS	(−)	(−)
9	*MSH6*	NM_000179.2	Exon 4	c.C926A	p.Ser309Tyr	Missense	Uncertain significance	(+)	(+)	(+)	(+)	MSS	(−)	(−)
10	*MSH6*	NM_000179.2	Exon 4	c.C926G	p.Ser309Cys	Missense	Uncertain significance	(+)	(+)	(+)	(+)	MSS	(−)	(−)
11	*MSH2*	NM_000251.2	Exon 1	c.C14A	p.Pro5Gln	Missense	Uncertain significance	(+)	(+)	(+)	(+)	MSS	(+)	(−)
12	*MSH6*	NM_000179.2	Exon 4	c.G1063A	p.Gly355Ser	Missense	Uncertain significance	(+)	(+)	(+)	(+)	MSS	(−)	(−)
13	*MSH6*	NM_000179.2	Exon 2	c.A335G	p.Asn112Ser	Missense	Uncertain significance	(+)	(+)	(+)	(+)	MSS	(−)	(−)
14	*MSH6*	NM_000179.2	Exon 5	c.C3260G	p.Pro1087Arg	Missense	Uncertain significance	(+)	(+)	(+)	(+)	MSS	(−)	(−)
15	*MSH6*	NM_000179.2	Exon 10	c.C4051G	p.His1351Asp	Missense	Uncertain significance	(+)	(+)	(+)	(+)	MSS	(−)	(−)
16	*MSH6*	NM_000179.2	Exon 4	c.C926G	p.Ser309Cys	Missense	Uncertain significance	(+)	(+)	(−)	(+)	MSI-H	(−)	(−)
17	*MSH6*	NM_000179.2	Exon 4	c.G1063A	p.Gly355Ser	Missense	Uncertain significance	(−)	(+)	(+)	(−)	MSI-H	(−)	(−)
18	*MSH6*	NM_000179.2	Exon 4	c.A1828G	p.Lys610Glu	Missense	Uncertain significance	(−)	(+)	(+)	(−)	NA	(−)	(−)
19	*MSH6*	NM_000179.2	Exon 4	c.A1937G	p.Lys646Arg	Missense	Uncertain significance	(−)	(+)	(+)	(−)	NA	(−)	(−)
20	*MSH2*	NM_000251.2	Exon 1	c.C14A	p.Pro5Gln	Missense	Uncertain significance	(+)	(+)	(+)	(+)	MSS	(−)	(−)

MMR: mismatch repair; IHC: immunohistochemistry; *MLH1*: mutL homolog 1; *MSH2*: mutS homolog 2; *MSH6*: mutS homolog 6; MSI: microsatellite instability; *PMS2*: postmeiotic segregation increased 2 (*S. cerevisiae*); MSI-H: high-frequency microsatellite instability; MSS: microsatellite stable; NA: not available

^a^All the mutations are heterozygous variants

### Accuracy of the screening methods

The family histories of all 111 patients were evaluated. Due to insufficient tumor tissues for examination, IHC was performed for only 102 (91.9%) patients, and MSI test was performed for 83 (74.8%) patients.

Based on their clinical characteristics, 14 patients (12.6%) were suspected as having LS-EC by the Amsterdam II criteria, and 2 of them also met the revised Bethesda guidelines. All of the 14 patients underwent IHC examination of MMR proteins, and 7 (50.0%) were found to have intact MMR genes; MSI test was performed for 8 of the 14 patients, and identified MSI-H (*MLH1* mutation) in 1 patient and MSS in 7 patients. However, NGS revealed that 12 (85.7%) of the 14 patients were misdiagnosed by the clinical criteria, and only 2 (14.3%) were confirmed to have LS-EC. On the other hand, of the 97 patients not suspected as having LS-EC by the clinical criteria, 4 were eventually diagnosed with LS-EC. The sensitivity, specificity, PPV, and NPV of the clinical criteria for identifying LS-EC were 33.3%, 88.6%, 14.3%, and 95.9%, respectively (Table 3).

**Table 3:** Accuracy of clinical diagnostic criteria, IHC of MMR proteins, and MSI test in screening Lynch syndrome-associated endometrial carcinoma

Screening method	NGS (cases)		Sensitivity (%)	Specificity (%)	PPV (%)	NPV (%)
	LS-EC	Non-LS-EC				
Total	6	105				
Clinical criteria			33.3	88.6	14.3	95.9
Met	2	12				
Unmet	4	93				
IHC			66.7	75.0	33.3	100
MMR-deficient	4	24				
MMR-intact	2	72				
MSI			100.0	89.9	33.3	100.0
Positive	4	8				
Negative	0	71				
IHC plus MSI			100.0	72.4	20.0	100.0
MMR-deficient or MSI-positive	6	24				
MMR-intact and MSI-negative	0	63				

Due to insufficient tumor tissues for examination, IHC was performed for only 102 patients, and MSI test was performed for 83 patients

IHC: immunohistochemistry; MMR: mismatch repair; MSI: microsatellite instability; NGS: next-generation sequencing; LS-EC: Lynch syndrome-associated endometrial carcinoma; NPV: negative predictive value; PPV: positive predictive value

**Fig. 2:**
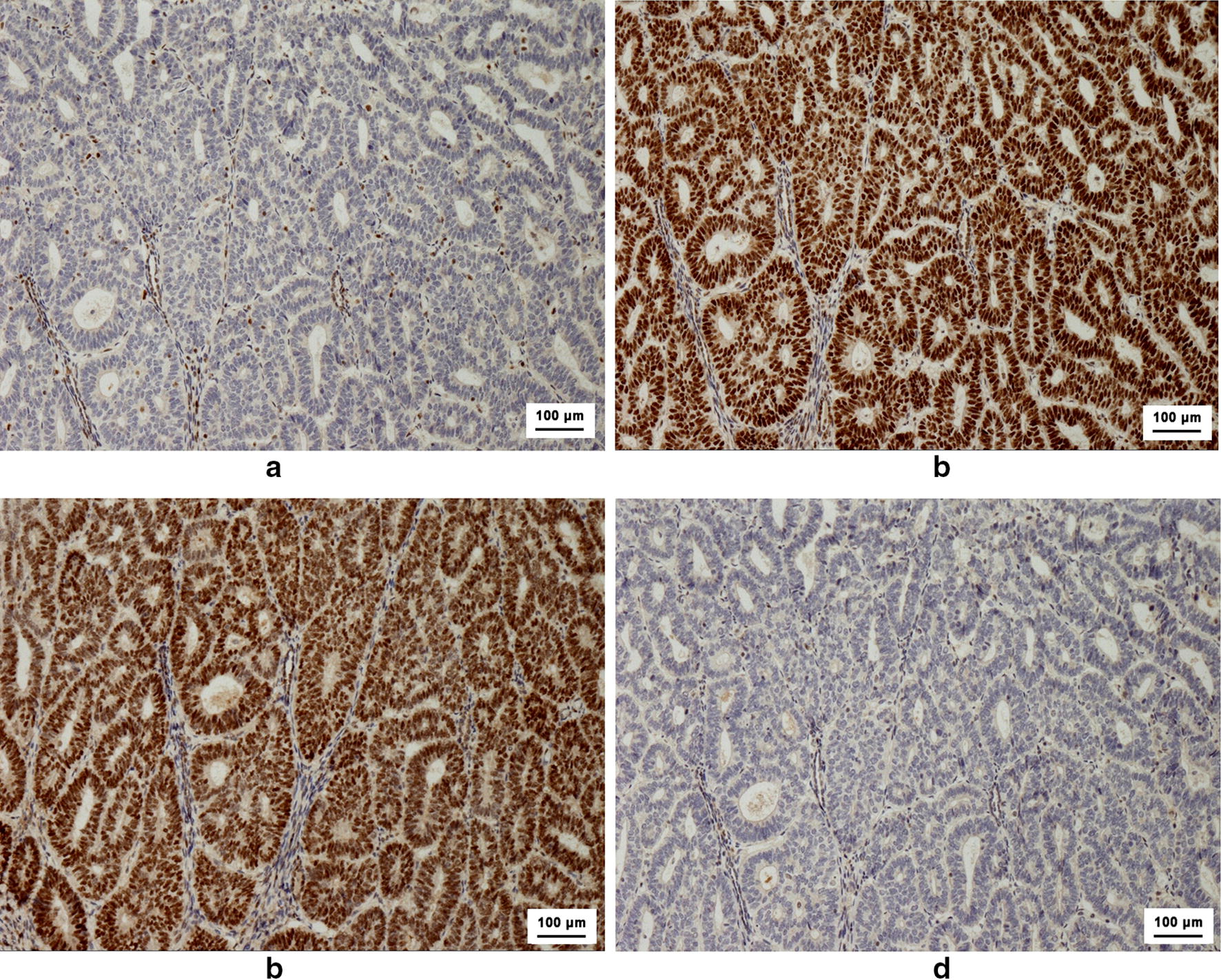
An example of immunohistochemistry (IHC) of mismatch repair (MMR) proteins from Case 4 in Table 2. This patient was 53 years at diagnosis and was confirmed to have grade 1 stage IA uterine endometrioid carcinoma. Two years before the diagnosis of endometrial cancer, she was diagnosed with early-stage colon cancer. Her father and daughter had a history of colon cancer and small intestine cancer, respectively. **a** Deficient expression of MutL homolog 1 (MLH1) protein in tumor tissues. The cytoplasm of almost all cancer cells had no staining in brown. **b** Normal expression of MutS homolog 2 (MSH2) protein in tumor tissues. The cytoplasm of cancer cells had homogeneous brown staining. **c** Normal expression of MutS homolog 6 (MSH6) protein in tumor tissues. The cytoplasm of cancer cells had homogeneous brown staining. **d** Deficient expression of PMS1 homolog 2 (PMS2) protein in tumor tissues. The cytoplasm of all cancer cells had no staining in brown

Among the 102 patients, IHC revealed that 28 (27.5%) were IHC MMR protein-deficient: 14 with both MLH1 and PMS2 protein deletion, 5 with both MSH2 and MSH6 deletion, 3 with MSH6 deletion, 3 with PMS2 deletion, and 1 each with MLH1 deletion, MSH2 deletion, and MLH1 and MSH6 deletion. The IHC MMR protein-deficient group was younger and had more advanced lesions than the IHC MMR protein-intact group, but these differences were not significant (Table 1). The sensitivity, specificity, PPV, and NPV of IHC of MMR proteins for identifying LS-EC were 66.7%, 75.0%, 14.3%, and 97.3%, respectively (Table 3). Among the 15 patients with MLH1 deletion, 3 had no hypermethylation of MLH1 promoter, and only one was confirmed to have LS-EC by NGS (Case 4 in Table 2 and Fig. 2). The 12 patients with MLH1 promoter hypermethylation harbored no deleterious mutations.

**Fig. 3:**
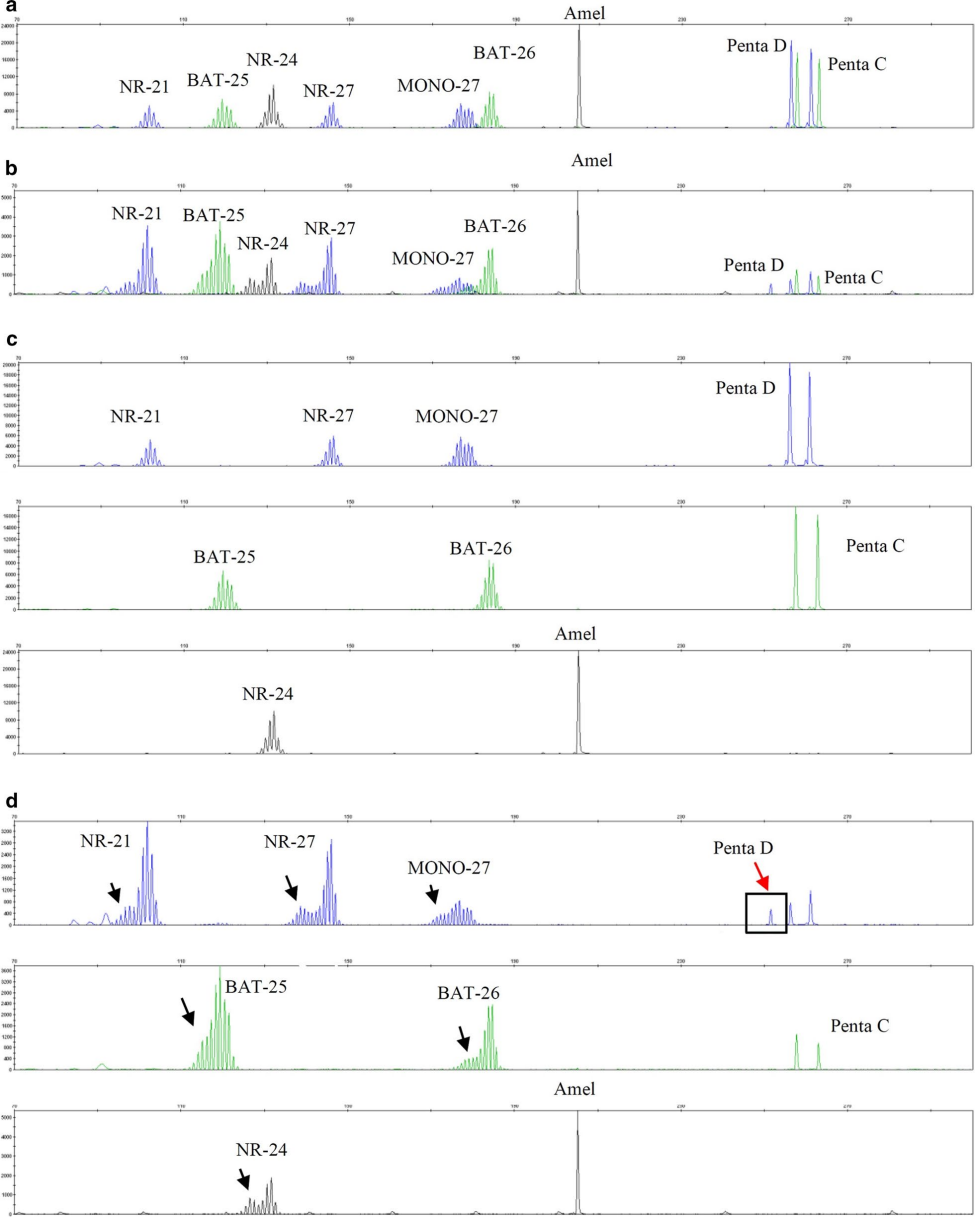
An example of MSI-H of Case 4 in Table 2. The introduction of this patient refers to the legend of Fig. 2. Electropherogram shows an allelic profile generated from a normal sample (**a**, **b**) or from a matching MSH6-deficient tumor sample (**c**, **d**) using the MSI detection kit. First, the sex-determination site Amel and the pentanucleotide markers Penta C and Penta D were analyzed. The Amel site and Penta C/Penta D were used to confirm that the tumor sample and the corresponding normal sample were from the same individual. Alleles found in the normal sample should also be present in the tumor sample; otherwise, there may be sample mixing or contamination. In this case, it is advisable to re-extract the sample for testing. The control sample (paraffin-embedded paracancerous tissue) is microsatellite stable (MSS). In this paraffin-embedded tumor tissue (**d**), a new allele appears compared to the normal sample (**b**) (new allele peak indicated by the arrow), that is, the mononucleotide repeat markers are unstable. Finally, the results were interpreted by comparing the numbers of changes in the mononucleotide repeat markers in the test samples. Note that additional pentanucleotide alleles of Penta C and Penta D may be present in some MSI-H samples (additional allele peaks indicated by the box are detailed in **d**)

MSI test of the 83 patients classified 12 (14.5%) as MSI-H and 71 (85.5%) as MSS. The MSI-positive group had higher gravidity than the MSI-negative group (*p *= 0.020) (Table 1). The sensitivity, specificity, PPV, and NPV of MSI test for identifying LS-EC were 100%, 89.9%, 33.3%, and 100%, respectively. An example of the MSI analysis is presented in Fig. 3.

There was no significant difference in sensitivity between IHC of MMR proteins and the clinical diagnostic criteria (*p *= 0.284) or between MSI test and the clinical diagnostic criteria (*p *= 0.071). For the combination of IHC of MMR proteins and MSI test, the sensitivity, specificity, PPV, and NPV were 100%, 72.4%, 20.0%, and 100%, respectively. Thus, the combination of IHC and MSI test had higher sensitivity than the clinical criteria (*p* = 0.030).

### Concordance between IHC of MMR proteins and MSI

A total of 77 patients had both IHC and MSI results available; 10 of them were MSI-H and MMR protein-deficient, and 63 were MSS and MMR protein-intact. Two patients were MSS and MMR protein-deficient, and 2 were MSI-H and MMR protein-intact. MSI test and IHC were highly concordant for LS-EC screening (73/77, 94.8%).

## Discussion

The present study reported the performance of universal screening and validation with NGS for LS in a cohort of surgically staged EC patients in China. In total, LS was confirmed in 6 (5.4%) EC patients by NGS. The prevalence of LS in EC patients is not low, suggesting that selective screening for LS among EC patients is unreasonable. Consistent with the previously reported LS prevalence of 8.5% in EC patients < 50 years old [26] and 5.9% in all-age EC patients [27], the present study revealed similar LS prevalence in a Chinese population of EC patients. LS was not diagnosed in any patient over 70 years of age in the present study, which may be related to the low prevalence of LS-EC in the general population and the small sample size of our cohort. However, the reported prevalence of LS-EC varied from 2.1% (21/1002) [19] to 3.9% (7/179) [20] and 6.6% (40/605) [12]. These differences were likely caused by the screening methods and study populations.

Although the protocol for screening EC patients for LS remains a subject of debate [28], the results in the present study suggest that universal molecular screening rather than using clinical criteria is necessary. However, the selection of appropriate methods and strategies is also debatable. Our data suggest that, for LS-EC, the clinical criteria should not be used alone in routine practice because of its low sensitivity and PPV. As over one-third of ECs in young women cannot be attributed to excess estrogen or hereditary syndromes [29], clinicopathological factors are not suitable for LS-EC screening [26], although some studies recommended selective screening based on the clinicopathological features because they can be examined inexpensively and noninvasively [30, 31]. There are several reasons that could explain the low PPV of the clinical criteria. *MSH6* mutation-associated EC is common in the elderly and patients with no family history of cancer, and the penetrance of *PMS2* mutation is lower than that of *MLH1* or *MSH2* mutation. Therefore, *MSH6* and *PMS2* mutations are especially likely to be missed in selective screening according to clinical criteria [32, 33]. Approximately 70% of LS patients did not meet the Amsterdam II criteria or revised Bethesda guidelines because the sizes of many families with mutations were too small or the cancer appeared late in life [34]. In addition, these criteria were formulated mainly for colorectal cancer, and EC was not adequately evaluated [32]. Mills et al. [15] reported that 57.1% of patients with LS would not be identified based on age and individual cancer history, and 28.6% would not be identified even with a complete family history. Overall, these problems limit the application of clinical criteria.

Our findings suggest that a combination of IHC of MMR proteins and MSI test for the screening of LS-EC would achieve favorable screening accuracy. This recommendation has been supported by recent reports and guidelines [19, 20, 29, 35, 36]. However, few studies used universal molecular screening and NGS for confirmation, which explained various and even inconsistent prevalence of LS-EC. More importantly, these reports did not include the data of Chinese population. Molecular screening methods had their own limitations. Given the high rate of *MSH6* mutations in patients with LS-EC and the low predictive value of MSI in identifying *MSH6* mutation-associated LS, IHC, rather than MSI test, has been suggested as the primary screening approach for LS in patients with EC [37]. However, IHC is limited by the high interobserver variability. Making a definitive diagnosis is difficult when the protein expression is weakly positive and heterogeneous [38, 39]. MSI also has disadvantages as a screening tool, such as its high cost and strict criteria for sequencing and interpretation. Indeed, according to the guideline recommendations [21, 35, 40] and the results of the present study, screening with only one method is insufficient, and combination screening is appropriate in the current clinical practice.

As previously reported [36] and observed in the present study, MSI test and IHC were highly concordant for EC screening. The discordant MMR protein-proficient/MSI-H cases (< 1%) may be explained by *MLH1* promoter hypermethylation or other unknown gene mutations. However, deviations in technique and interpretation may also be partially responsible for this discordance. As our data suggested, hypermethylation is an important indication of conducting NGS in MLH1-negative patients. In the present study, 3 (20.0%) of 15 MLH1-negative patients had no hypermethylation, and 1 of them was confirmed to have LS-EC, which was consistent with previous reports [15, 20].

The current study has some limitations. The significant association between EC risk and family history regardless of proband MMR status and after the exclusion of probands carrying pathogenic MMR variants indicated that MMR genes could not fully explain familial EC risk [41]. Thus, continued research on familial EC is essential. Polygenic interactions have been suggested to be the most likely cause of cancer development in LS patients [42]. Hence, multigene panel test in a large cohort is ongoing at our institute to more definitively determine the prevalence of hereditary EC in China. Additionally, the limited sample size of the present study probably limits the generalizability of our findings. Finally, a cost-effectiveness analysis is lacking due to the limited sample size. In the present study, the costs of molecular screening methods and NGS were much lower than previously reported [20, 43]. A comprehensive analysis of cost-effectiveness would aid in optimizing screening strategies for LS [44, 45]. Routine LS screening in patients ≤ 70 years of age has been demonstrated to be a cost-effective strategy [20, 46]. Key determinants of whether screening is cost-effective are the accuracy of tumor-based tests, the risk of cancer without surveillance, the number of relatives identified for cascade test, the effectiveness of colonoscopy surveillance, and patient acceptance of genetic test [47].

## Conclusions

Our findings showed that for identifying LS in women presenting with EC, screening with a combination of IHC of MMR proteins and MSI test was superior to the clinical criteria alone. MSI test and IHC were found to be highly concordant for EC screening. Although only six cases of LS-EC were identified in the present study, universal screening and validation in unselected patients would provide substantial evidence to inform clinical decision making between patients and physicians.

## Data Availability

The datasets used in this study are available from the corresponding author on reasonable request.

## Abbreviations

LS: Lynch syndrome
DNA: deoxyribonucleic acid
MMR: mismatch repair
LS-EC: LS-associated endometrial carcinoma
EC: endometrial carcinoma
MLH1: MutL homolog 1
MSH2: MutS homolog 2
MSH6: MutS homolog 6
PMS2: PMS1 homolog 2
EPCAM: epithelial cell adhesion molecule
NCCN: National Comprehensive Cancer Network
IHC: immunohistochemical
MSI: microsatellite instability
AGO: Arbeitsgemeinschaft für Gynäkologische Onkologie (Austria)
CAMS: Chinese Academy of Medical Sciences
MS-MLPA: methylation-specific multiplex ligation-dependent probe amplification
PCR: polymerase chain reaction
MSI-H: high-frequency MSI
MSI-L: low-frequency MSI
MSS: microsatellite stable
tPAS: TILLING PCR amplifier sequence
qPCR: quantitative polymerase chain reaction
SNPs: single-nucleotide polymorphisms
ACMG: American College for Medical Genetics and Genomics
NGS: next-generation sequencing
SPSS: Statistical Product and Service Solutions
SDs: standard deviations
FIGO: International Federation of Gynecology and Obstetrics
BMI: body mass index
PPV: positive predictive value
NPV: negative predictive value

## References

[1] Moller P, Seppala TT, Bernstein I, Holinski-Feder E, Sala P, Gareth Evans D (2018). Cancer risk and survival in path_MMR carriers by gene and gender up to 75 years of age: a report from the Prospective Lynch syndrome database. Gut.

[2] Hampel H, Bennett RL, Buchanan A, Pearlman R, Wiesner GL (2015). A practice guideline from the American College of Medical Genetics and Genomics and the National Society of Genetic Counselors: referral indications for cancer predisposition assessment. Genet Med.

[3] Boilesen AE, Bisgaard ML, Bernstein I (2008). Risk of gynecologic cancers in Danish hereditary non-polyposis colorectal cancer families. Acta Obstet Gynecol Scand.

[4] Watson P, Vasen HFA, Mecklin JP, Bernstein I, Aarnio M, Jarvinen HJ (2008). The risk of extra-colonic, extra-endometrial cancer in the Lynch syndrome. Int J Cancer.

[5] Tafe LJ, Riggs ER, Tsongalis GJ (2014). Lynch syndrome presenting as endometrial cancer. Clin Chem.

[6] Wang Y, Li J, Cragun J, Hatch K, Chambers SK, Zheng W (2013). Lynch syndrome related endometrial cancer: clinical significance beyond the endometrium. J Hematol Oncol.

[7] Moller P, Seppala T, Bernstein I, Holinski-Feder E, Sala P, Evans DG (2017). Cancer incidence and survival in Lynch syndrome patients receiving colonoscopic and gynaecological surveillance: first report from the prospective Lynch syndrome database. Gut.

[8] Lynch HT, Boland CR, Gong G, Shaw TG, Lynch PM, Fodde R (2006). Phenotypic and genotypic heterogeneity in the Lynch syndrome: diagnostic, surveillance and management implications. Eur J Hum Genet.

[9] Sinicrope FA (2018). Lynch syndrome—associated colorectal cancer. N Engl J Med.

[10] Hampel H, Pearlman R, Beightol M, Zhao W, Jones D, Frankel WL (2018). Assessment of tumor sequencing as a replacement for Lynch syndrome screening and current molecular tests for patients with colorectal cancer. JAMA Oncol.

[11] Clarke BA, Cooper K (2012). Identifying Lynch syndrome in patients with endometrial carcinoma: shortcomings of morphologic and clinical schemas. Adv Anat Pathol.

[12] Mills AM, Liou S, Ford JM, Berek JS, Pai RK, Longacre TA (2014). Lynch syndrome screening should be considered for all patients with newly diagnosed endometrial cancer. Am J Surg Pathol.

[13] Vasen HF, Watson P, Mecklin JP, Lynch HT (1999). New clinical criteria for hereditary nonpolyposis colorectal cancer (HNPCC, Lynch syndrome) proposed by the International Collaborative group on HNPCC. Gastroenterology.

[14] Umar A, Boland CR, Terdiman JP, Syngal S, de la Chapelle A, Ruschoff J (2004). Revised Bethesda Guidelines for hereditary nonpolyposis colorectal cancer (Lynch syndrome) and microsatellite instability. J Natl Cancer Inst.

[15] Mills AM, Sloan EA, Thomas M, Modesitt SC, Stoler MH, Atkins KA (2016). Clinicopathologic comparison of Lynch syndrome-associated and “Lynch-like” endometrial carcinomas identified on universal screening using mismatch repair protein immunohistochemistry. Am J Surg Pathol.

[16] Ryan P, Mulligan AM, Aronson M, Ferguson SE, Bapat B, Semotiuk K (2012). Comparison of clinical schemas and morphologic features in predicting Lynch syndrome in mutation-positive patients with endometrial cancer encountered in the context of familial gastrointestinal cancer registries. Cancer.

[17] Qian CN (2017). At-home cancer screening: a solution for China and other developing countries with a large population and limited number of healthcare practitioners. Chin J Cancer.

[18] NCCN Clinical Practice Guidelines in Oncology (NCCN Guidelines^®^). Uterine neoplasms. Version 1. 2019. https://www.nccn.org/professionals/physician_gls/pdf/uterine.pdf. Accessed 15 Jan 2019.

[19] Goodfellow PJ, Billingsley CC, Lankes HA, Ali S, Cohn DE, Broaddus RJ (2015). Combined microsatellite instability, MLH1 methylation analysis, and immunohistochemistry for Lynch syndrome screening in endometrial cancers from GOG210: an NRG Oncology and Gynecologic Oncology Group Study. J Clin Oncol.

[20] Goverde A, Spaander MC, van Doorn HC, Dubbink HJ, van den Ouweland AM, Tops CM (2016). Cost-effectiveness of routine screening for Lynch syndrome in endometrial cancer patients up to 70 years of age. Gynecol Oncol.

[21] Zeimet AG, Mori H, Petru E, Polterauer S, Reinthaller A, Schauer C (2017). AGO Austria recommendation on screening and diagnosis of Lynch syndrome (LS). Arch Gynecol Obstet.

[22] Zhou JY, Zhang L, Wei LH, Wang JL (2016). Endometrial carcinoma-related genetic factors: application to research and clinical practice in China. BJOG.

[23] van Lier MG, Wagner A, van Leerdam ME, Biermann K, Kuipers EJ, Steyerberg EW (2010). A review on the molecular diagnostics of Lynch syndrome: a central role for the pathology laboratory. J Cell Mol Med.

[24] Rodriguez-Bigas MA, Boland CR, Hamilton SR, Henson DE, Jass JR, Khan PM (1997). A National Cancer Institute workshop on hereditary nonpolyposis colorectal cancer syndrome: meeting highlights and bethesda guidelines. J Natl Cancer Inst.

[25] Richards S, Aziz N, Bale S, Bick D, Das S, Gastier-Foster J (2015). Standards and guidelines for the interpretation of sequence variants: a joint consensus recommendation of the American College of Medical Genetics and Genomics and the Association for Molecular Pathology. Genet Med.

[26] Anagnostopoulos A, McKay VH, Cooper I, Campbell F, Greenhalgh L, Kirwan J (2017). Identifying Lynch syndrome in women presenting with endometrial carcinoma under the age of 50 years. Int J Gynecol Cancer.

[27] Ferguson SE, Aronson M, Pollett A, Eiriksson LR, Oza AM, Gallinger S (2014). Performance characteristics of screening strategies for Lynch syndrome in unselected women with newly diagnosed endometrial cancer who have undergone universal germline mutation testing. Cancer.

[28] Lynch HT, Snyder CL, Shaw TG, Heinen CD, Hitchins MP (2015). Milestones of Lynch syndrome: 1895–2015. Nat Rev Cancer.

[29] Burleigh A, Talhouk A, Gilks CB, McAlpine JN (2015). Clinical and pathological characterization of endometrial cancer in young women: identification of a cohort without classical risk factors. Gynecol Oncol.

[30] Moline J, Mahdi H, Yang B, Biscotti C, Roma AA, Heald B (2013). Implementation of tumor testing for Lynch syndrome in endometrial cancers at a large academic medical center. Gynecol Oncol.

[31] Rabban JT, Calkins SM, Karnezis AN, Grenert JP, Blanco A, Crawford B (2014). Association of tumor morphology with mismatch-repair protein status in older endometrial cancer patients: implications for universal versus selective screening strategies for Lynch syndrome. Am J Surg Pathol.

[32] Batte BA, Bruegl AS, Daniels MS, Ring KL, Dempsey KM, Djordjevic B (2014). Consequences of universal MSI/IHC in screening endometrial cancer patients for lynch syndrome. Gynecol Oncol.

[33] Moline J, Eng C (2014). Equality in Lynch syndrome screening: why should we hold patients with endometrial cancer to a different standard?. J Clin Oncol.

[34] Cohen SA, Leininger A (2014). The genetic basis of Lynch syndrome and its implications for clinical practice and risk management. Appl Clin Genet.

[35] NCCN Clinical Practice Guidelines in Oncology (NCCN Guidelines^®^). Genetic/Familial High-risk Assessment: Colorectoral. Version 1. 2018. https://www.nccn.org/professionals/physician_gls/pdf/genetics_colon.pdf. Accessed 12 July 2018.

[36] Stelloo E, Jansen AM, Osse EM, Nout RA, Creutzberg CL, Ruano D (2017). Practical guidance for mismatch repair-deficiency testing in endometrial cancer. Ann Oncol.

[37] Resnick KE, Hampel H, Fishel R, Cohn DE (2009). Current and emerging trends in Lynch syndrome identification in women with endometrial cancer. Gynecol Oncol.

[38] Shia J, Holck S, Depetris G, Greenson JK, Klimstra DS (2013). Lynch syndrome-associated neoplasms: a discussion on histopathology and immunohistochemistry. Fam Cancer.

[39] Kato A, Sato N, Sugawara T, Takahashi K, Kito M, Makino K (2016). Isolated loss of PMS2 immunohistochemical expression is frequently caused by heterogenous MLH1 promoter hypermethylation in Lynch syndrome screening for endometrial cancer patients. Am J Surg Pathol.

[40] Vasen HF, Blanco I, Aktan-Collan K, Gopie JP, Alonso A, Aretz S (2013). Revised guidelines for the clinical management of Lynch syndrome (HNPCC): recommendations by a group of European experts. Gut.

[41] Johnatty SE, Tan YY, Buchanan DD, Bowman M, Walters RJ, Obermair A (2017). Family history of cancer predicts endometrial cancer risk independently of Lynch syndrome: implications for genetic counselling. Gynecol Oncol.

[42] Talseth-Palmer BA, Bauer DC, Sjursen W, Evans TJ, McPhillips M, Proietto A (2016). Targeted next-generation sequencing of 22 mismatch repair genes identifies Lynch syndrome families. Cancer Med.

[43] Usha L, Dewdney SB, Buckingham LE (2016). Tumor screening and DNA testing in the diagnosis of Lynch Syndrome. JAMA.

[44] Barzi A, Sadeghi S, Kattan MW, Meropol NJ (2015). Comparative effectiveness of screening strategies for Lynch syndrome. J Natl Cancer Inst.

[45] Di Marco M, DAndrea E, Panic N, Baccolini V, Migliara G, Marzuillo C (2018). Which Lynch syndrome screening programs could be implemented in the “real world”? A systematic review of economic evaluations. Genet Med.

[46] Resnick K, Straughn JM, Backes F, Hampel H, Matthews KS, Cohn DE (2009). Lynch syndrome screening strategies among newly diagnosed endometrial cancer patients. Obstet Gynecol.

[47] Snowsill T, Coelho H, Huxley N, Jones-Hughes T, Briscoe S, Frayling IM (2017). Molecular testing for Lynch syndrome in people with colorectal cancer: systematic reviews and economic evaluation. Health Technol Assess.

